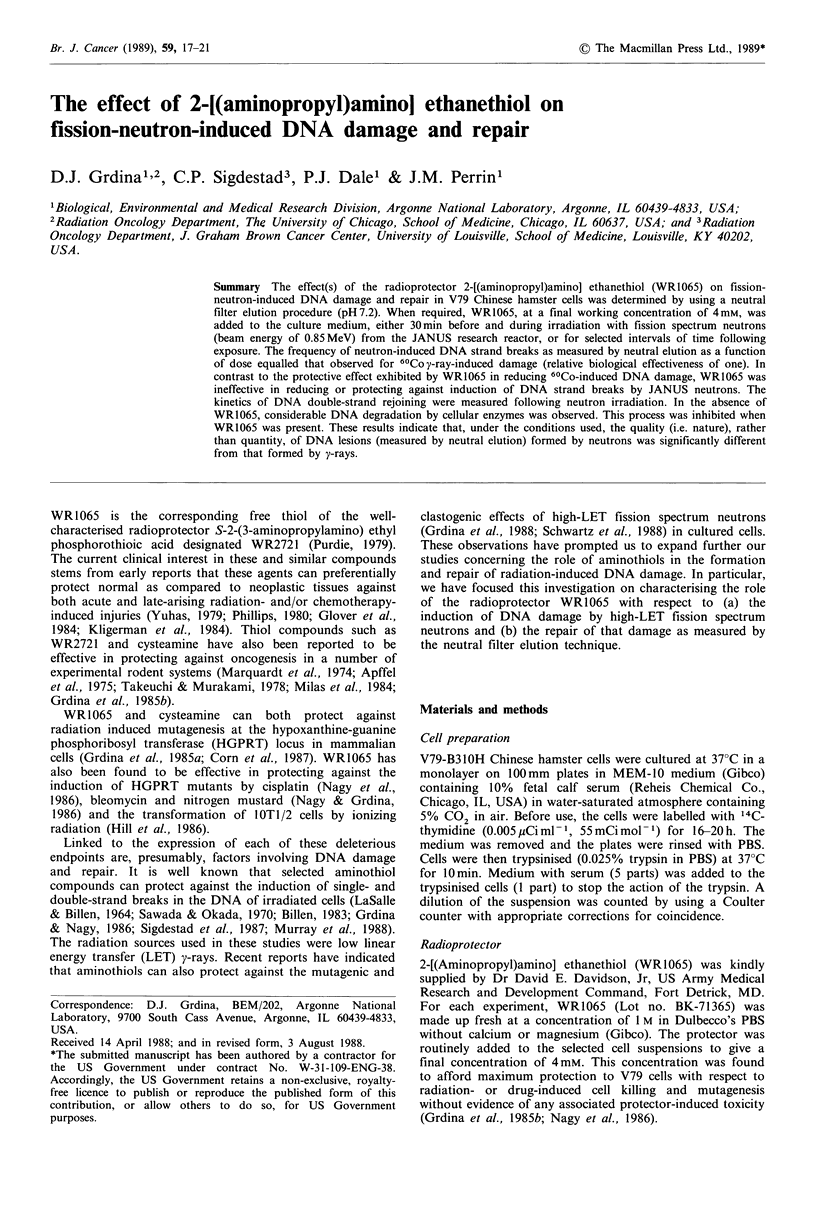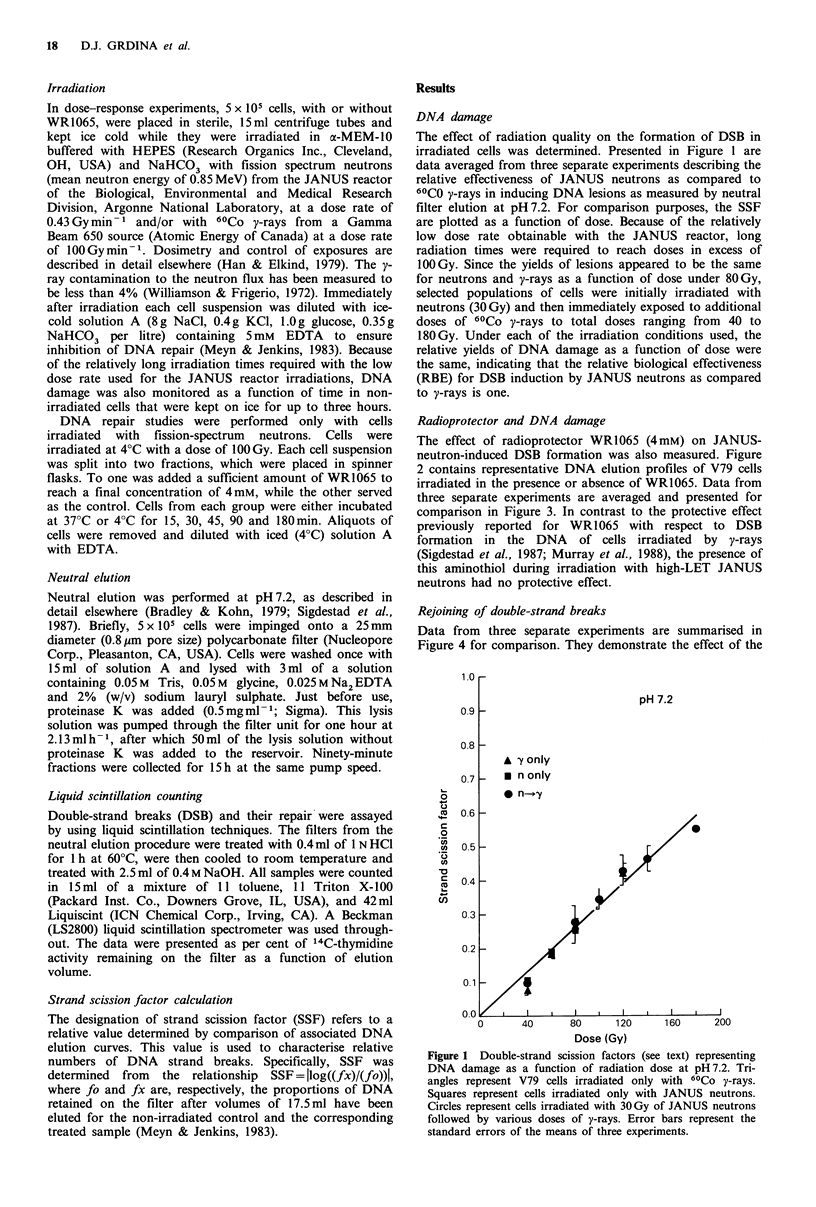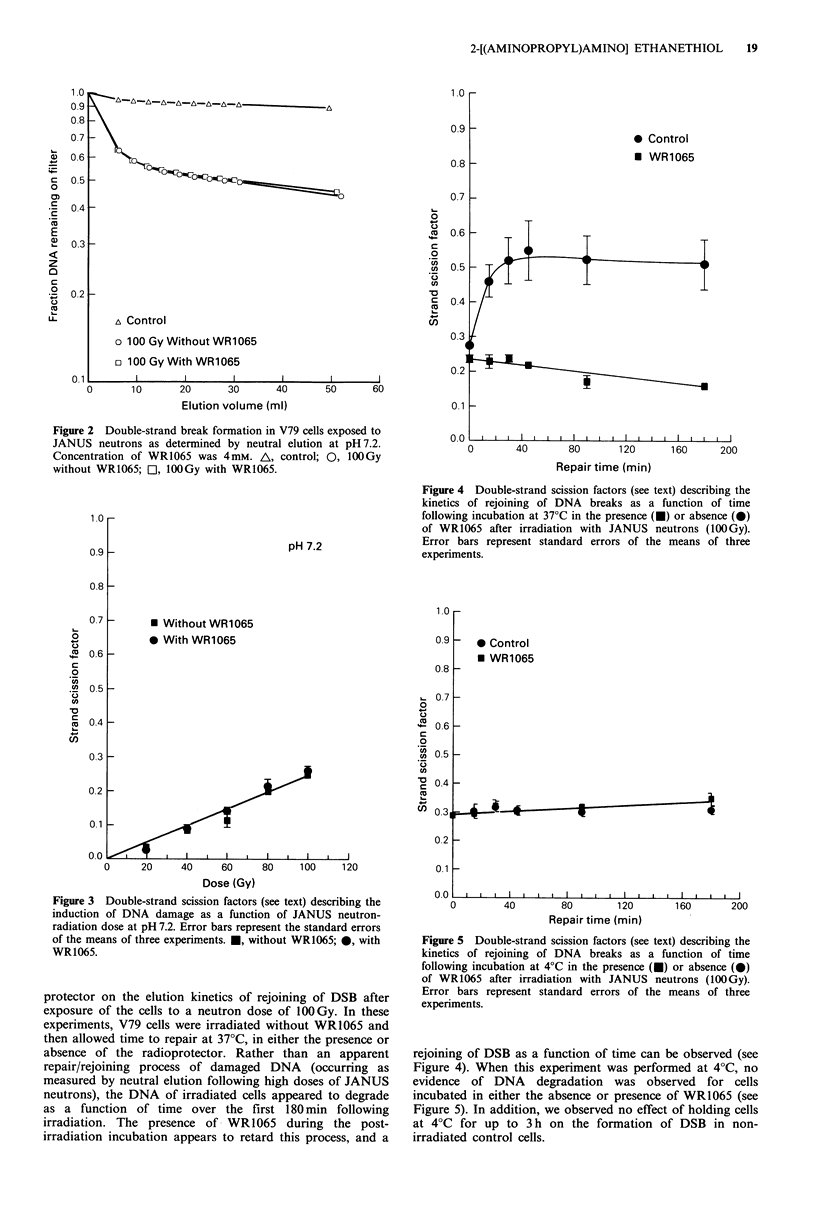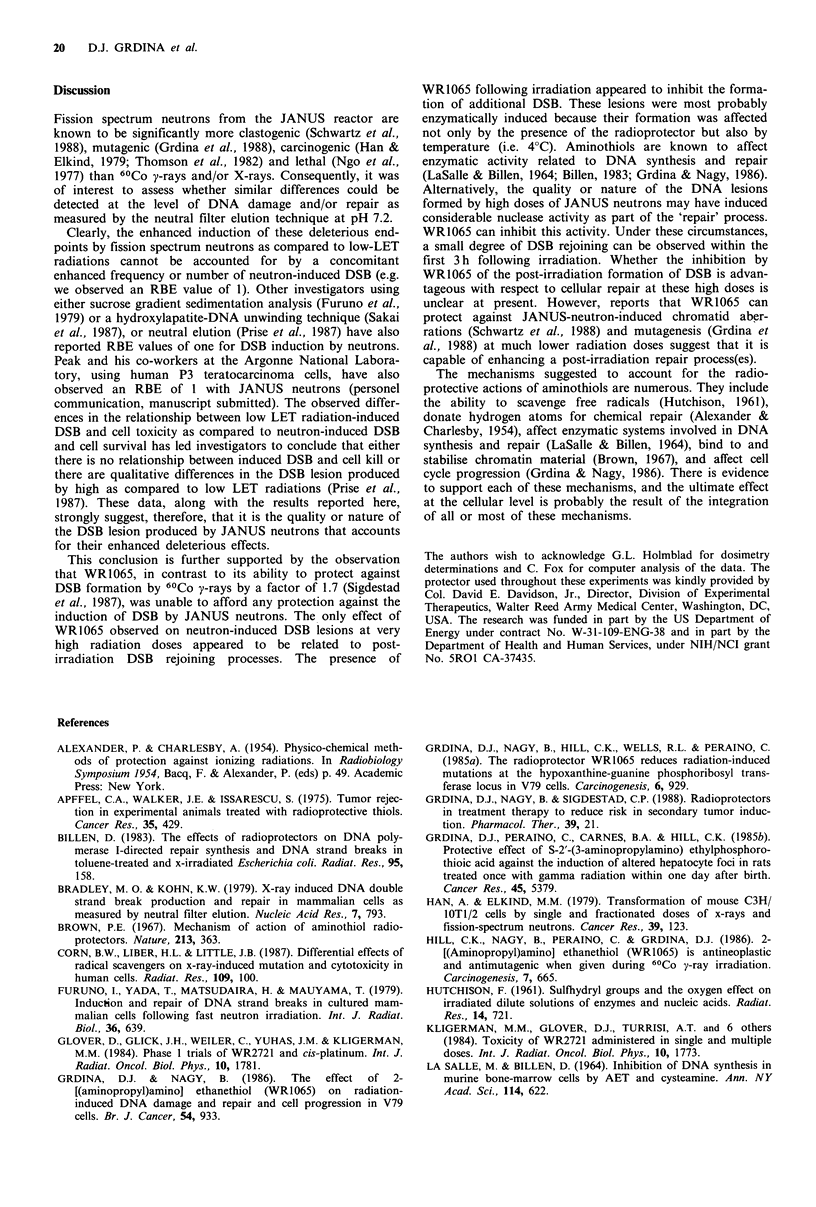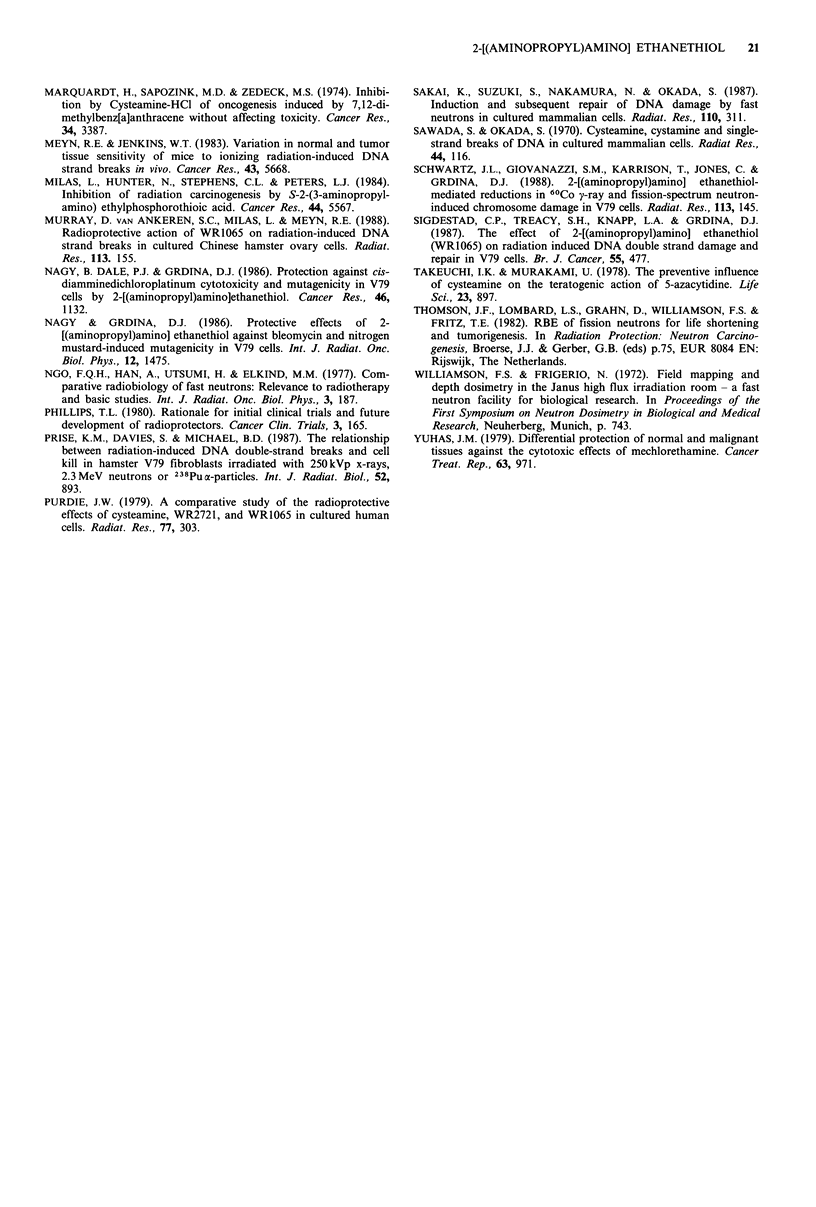# The effect of 2-[(aminopropyl)amino] ethanethiol on fission-neutron-induced DNA damage and repair.

**DOI:** 10.1038/bjc.1989.5

**Published:** 1989-01

**Authors:** D. J. Grdina, C. P. Sigdestad, P. J. Dale, J. M. Perrin

**Affiliations:** Biological, Environmental and Medical Research Division, Argonne National Laboratory, IL 60439-4833.

## Abstract

The effect(s) of the radioprotector 2-[(aminopropyl)amino] ethanethiol (WR 1065) on fission-neutron-induced DNA damage and repair in V79 Chinese hamster cells was determined by using a neutral filter elution procedure (pH 7.2). When required, WR1065, at a final working concentration of 4 mM, was added to the culture medium, either 30 min before and during irradiation with fission spectrum neutrons (beam energy of 0.85 MeV) from the JANUS research reactor, or for selected intervals of time following exposure. The frequency of neutron-induced DNA strand breaks as measured by neutral elution as a function of dose equalled that observed for 60Co gamma-ray-induced damage (relative biological effectiveness of one). In contrast to the protective effect exhibited by WR1065 in reducing 60Co-induced DNA damage, WR1065 was ineffective in reducing or protecting against induction of DNA strand breaks by JANUS neutrons. The kinetics of DNA double-strand rejoining were measured following neutron irradiation. In the absence of WR1065, considerable DNA degradation by cellular enzymes was observed. This process was inhibited when WR1065 was present. These results indicate that, under the conditions used, the quality (i.e. nature), rather than quantity, of DNA lesions (measured by neutral elution) formed by neutrons was significantly different from that formed by gamma-rays.


*Br. J. Cancer* (1989), **59**, 17–21 © The Macmillan Press Ltd., 1989*

### The effect of 2-[(aminopropyl)amino] ethanethiol on fission-neutron-induced DNA damage and repair

D.J. Grdina[1,2], C.P. Sigdestad[3], P.J. Dale[1] & J.M. Perrin[1]

[1]*Biological, Environmental and Medical Research Division, Argonne National Laboratory, Argonne, IL 60439-4833, USA;* [2]*Radiation Oncology Department, The University of Chicago, School of Medicine, Chicago, IL 60637, USA; and* [3]*Radiation Oncology Department, J. Graham Brown Cancer Center, University of Louisville, School of Medicine, Louisville, KY 40202, USA.*

**Summary** The effect(s) of the radioprotector 2-[(aminopropyl)amino] ethanethiol (WR1065) on fission-neutron-induced DNA damage and repair in V79 Chinese hamster cells was determined by using a neutral filter elution procedure (pH 7.2). When required, WR1065, at a final working concentration of 4 mM, was added to the culture medium, either 30 min before and during irradiation with fission spectrum neutrons (beam energy of 0.85 MeV) from the JANUS research reactor, or for selected intervals of time following exposure. The frequency of neutron-induced DNA strand breaks as measured by neutral elution as a function of dose equalled that observed for $^{60}$Co $\gamma$-ray-induced damage (relative biological effectiveness of one). In contrast to the protective effect exhibited by WR1065 in reducing $^{60}$Co-induced DNA damage, WR1065 was ineffective in reducing or protecting against induction of DNA strand breaks by JANUS neutrons. The kinetics of DNA double-strand rejoining were measured following neutron irradiation. In the absence of WR1065, considerable DNA degradation by cellular enzymes was observed. This process was inhibited when WR1065 was present. These results indicate that, under the conditions used, the quality (i.e. nature), rather than quantity, of DNA lesions (measured by neutral elution) formed by neutrons was significantly different from that formed by $\gamma$-rays.

WR1065 is the corresponding free thiol of the well-characterised radioprotector *S*-2-(3-aminopropylamino) ethyl phosphorothioic acid designated WR2721 (Purdie, 1979). The current clinical interest in these and similar compounds stems from early reports that these agents can preferentially protect normal as compared to neoplastic tissues against both acute and late-arising radiation- and/or chemotherapy-induced injuries (Yuhas, 1979; Phillips, 1980; Glover *et al.*, 1984; Kligerman *et al.*, 1984). Thiol compounds such as WR2721 and cysteamine have also been reported to be effective in protecting against oncogenesis in a number of experimental rodent systems (Marquardt *et al.*, 1974; Apffel *et al.*, 1975; Takeuchi & Murakami, 1978; Milas *et al.*, 1984; Grdina *et al.*, 1985*b*).

WR1065 and cysteamine can both protect against radiation induced mutagenesis at the hypoxanthine-guanine phosphoribosyl transferase (HGPRT) locus in mammalian cells (Grdina *et al.*, 1985*a*; Corn *et al.*, 1987). WR1065 has also been found to be effective in protecting against the induction of HGPRT mutants by cisplatin (Nagy *et al.*, 1986), bleomycin and nitrogen mustard (Nagy & Grdina, 1986) and the transformation of 10T1/2 cells by ionizing radiation (Hill *et al.*, 1986).

Linked to the expression of each of these deleterious endpoints are, presumably, factors involving DNA damage and repair. It is well known that selected aminothiol compounds can protect against the induction of single- and double-strand breaks in the DNA of irradiated cells (LaSalle & Billen, 1964; Sawada & Okada, 1970; Billen, 1983; Grdina & Nagy, 1986; Sigdestad *et al.*, 1987; Murray *et al.*, 1988). The radiation sources used in these studies were low linear energy transfer (LET) $\gamma$-rays. Recent reports have indicated that aminothiols can also protect against the mutagenic and clastogenic effects of high-LET fission spectrum neutrons (Grdina *et al.*, 1988; Schwartz *et al.*, 1988) in cultured cells. These observations have prompted us to expand further our studies concerning the role of aminothiols in the formation and repair of radiation-induced DNA damage. In particular, we have focused this investigation on characterising the role of the radioprotector WR1065 with respect to (a) the induction of DNA damage by high-LET fission spectrum neutrons and (b) the repair of that damage as measured by the neutral filter elution technique.

### Materials and methods

### *Cell preparation*

V79-B310H Chinese hamster cells were cultured at 37°C in a monolayer on 100 mm plates in MEM-10 medium (Gibco) containing 10% fetal calf serum (Reheis Chemical Co., Chicago, IL, USA) in water-saturated atmosphere containing 5% $CO_2$ in air. Before use, the cells were labelled with $^{14}$C-thymidine (0.005 $\mu$Ci ml$^{-1}$, 55 mCi mol$^{-1}$) for 16–20 h. The medium was removed and the plates were rinsed with PBS. Cells were then trypsinised (0.025% trypsin in PBS) at 37°C for 10 min. Medium with serum (5 parts) was added to the trypsinised cells (1 part) to stop the action of the trypsin. A dilution of the suspension was counted by using a Coulter counter with appropriate corrections for coincidence.

### *Radioprotector*

2-[(Aminopropyl)amino] ethanethiol (WR1065) was kindly supplied by Dr David E. Davidson, Jr, US Army Medical Research and Development Command, Fort Detrick, MD. For each experiment, WR1065 (Lot no. BK-71365) was made up fresh at a concentration of 1 M in Dulbecco's PBS without calcium or magnesium (Gibco). The protector was routinely added to the selected cell suspensions to give a final concentration of 4 mM. This concentration was found to afford maximum protection to V79 cells with respect to radiation- or drug-induced cell killing and mutagenesis without evidence of any associated protector-induced toxicity (Grdina *et al.*, 1985*b*; Nagy *et al.*, 1986).

Correspondence: D.J. Grdina, BEM/202, Argonne National Laboratory, 9700 South Cass Avenue, Argonne, IL 60439-4833, USA.
Received 14 April 1988; and in revised form, 3 August 1988.
*The submitted manuscript has been authored by a contractor for the US Government under contract No. W-31-109-ENG-38. Accordingly, the US Government retains a non-exclusive, royalty-free licence to publish or reproduce the published form of this contribution, or allow others to do so, for US Government purposes.

*Irradiation*

In dose–response experiments, $5 \times 10^5$ cells, with or without WR1065, were placed in sterile, 15 ml centrifuge tubes and kept ice cold while they were irradiated in α-MEM-10 buffered with HEPES (Research Organics Inc., Cleveland, OH, USA) and $NaHCO_3$ with fission spectrum neutrons (mean neutron energy of 0.85 MeV) from the JANUS reactor of the Biological, Environmental and Medical Research Division, Argonne National Laboratory, at a dose rate of 0.43 Gy min$^{-1}$ and/or with $^{60}$Co γ-rays from a Gamma Beam 650 source (Atomic Energy of Canada) at a dose rate of 100 Gy min$^{-1}$. Dosimetry and control of exposures are described in detail elsewhere (Han & Elkind, 1979). The γ-ray contamination to the neutron flux has been measured to be less than 4% (Williamson & Frigerio, 1972). Immediately after irradiation each cell suspension was diluted with ice-cold solution A (8 g NaCl, 0.4 g KCl, 1.0 g glucose, 0.35 g $NaHCO_3$ per litre) containing 5 mM EDTA to ensure inhibition of DNA repair (Meyn & Jenkins, 1983). Because of the relatively long irradiation times required with the low dose rate used for the JANUS reactor irradiations, DNA damage was also monitored as a function of time in non-irradiated cells that were kept on ice for up to three hours.

DNA repair studies were performed only with cells irradiated with fission-spectrum neutrons. Cells were irradiated at 4°C with a dose of 100 Gy. Each cell suspension was split into two fractions, which were placed in spinner flasks. To one was added a sufficient amount of WR1065 to reach a final concentration of 4 mM, while the other served as the control. Cells from each group were either incubated at 37°C or 4°C for 15, 30, 45, 90 and 180 min. Aliquots of cells were removed and diluted with iced (4°C) solution A with EDTA.

*Neutral elution*

Neutral elution was performed at pH 7.2, as described in detail elsewhere (Bradley & Kohn, 1979; Sigdestad *et al.*, 1987). Briefly, $5 \times 10^5$ cells were impinged onto a 25 mm diameter (0.8 μm pore size) polycarbonate filter (Nucleopore Corp., Pleasanton, CA, USA). Cells were washed once with 15 ml of solution A and lysed with 3 ml of a solution containing 0.05 M Tris, 0.05 M glycine, 0.025 M $Na_2$EDTA and 2% (w/v) sodium lauryl sulphate. Just before use, proteinase K was added (0.5 mg ml$^{-1}$; Sigma). This lysis solution was pumped through the filter unit for one hour at 2.13 ml h$^{-1}$, after which 50 ml of the lysis solution without proteinase K was added to the reservoir. Ninety-minute fractions were collected for 15 h at the same pump speed.

*Liquid scintillation counting*

Double-strand breaks (DSB) and their repair were assayed by using liquid scintillation techniques. The filters from the neutral elution procedure were treated with 0.4 ml of 1 N HCl for 1 h at 60°C, were then cooled to room temperature and treated with 2.5 ml of 0.4 M NaOH. All samples were counted in 15 ml of a mixture of 1 l toluene, 1 l Triton X-100 (Packard Inst. Co., Downers Grove, IL, USA), and 42 ml Liquiscint (ICN Chemical Corp., Irving, CA). A Beckman (LS2800) liquid scintillation spectrometer was used throughout. The data were presented as per cent of $^{14}$C-thymidine activity remaining on the filter as a function of elution volume.

*Strand scission factor calculation*

The designation of strand scission factor (SSF) refers to a relative value determined by comparison of associated DNA elution curves. This value is used to characterise relative numbers of DNA strand breaks. Specifically, SSF was determined from the relationship $SSF = |\log((fx)/(fo))|$, where $fo$ and $fx$ are, respectively, the proportions of DNA retained on the filter after volumes of 17.5 ml have been eluted for the non-irradiated control and the corresponding treated sample (Meyn & Jenkins, 1983).

### Results

*DNA damage*

The effect of radiation quality on the formation of DSB in irradiated cells was determined. Presented in Figure 1 are data averaged from three separate experiments describing the relative effectiveness of JANUS neutrons as compared to $^{60}$C0 γ-rays in inducing DNA lesions as measured by neutral filter elution at pH 7.2. For comparison purposes, the SSF are plotted as a function of dose. Because of the relatively low dose rate obtainable with the JANUS reactor, long radiation times were required to reach doses in excess of 100 Gy. Since the yields of lesions appeared to be the same for neutrons and γ-rays as a function of dose under 80 Gy, selected populations of cells were initially irradiated with neutrons (30 Gy) and then immediately exposed to additional doses of $^{60}$Co γ-rays to total doses ranging from 40 to 180 Gy. Under each of the irradiation conditions used, the relative yields of DNA damage as a function of dose were the same, indicating that the relative biological effectiveness (RBE) for DSB induction by JANUS neutrons as compared to γ-rays is one.

*Radioprotector and DNA damage*

The effect of radioprotector WR1065 (4 mM) on JANUS-neutron-induced DSB formation was also measured. Figure 2 contains representative DNA elution profiles of V79 cells irradiated in the presence or absence of WR1065. Data from three separate experiments are averaged and presented for comparison in Figure 3. In contrast to the protective effect previously reported for WR1065 with respect to DSB formation in the DNA of cells irradiated by γ-rays (Sigdestad *et al.*, 1987; Murray *et al.*, 1988), the presence of this aminothiol during irradiation with high-LET JANUS neutrons had no protective effect.

*Rejoining of double-strand breaks*

Data from three separate experiments are summarised in Figure 4 for comparison. They demonstrate the effect of the

**Figure 1** Double-strand scission factors (see text) representing DNA damage as a function of radiation dose at pH 7.2. Triangles represent V79 cells irradiated only with $^{60}$Co γ-rays. Squares represent cells irradiated only with JANUS neutrons. Circles represent cells irradiated with 30 Gy of JANUS neutrons followed by various doses of γ-rays. Error bars represent the standard errors of the means of three experiments.

**Figure 2** Double-strand break formation in V79 cells exposed to JANUS neutrons as determined by neutral elution at pH 7.2. Concentration of WR1065 was 4 mM. △, control; ○, 100 Gy without WR1065; □, 100 Gy with WR1065.

**Figure 3** Double-strand scission factors (see text) describing the induction of DNA damage as a function of JANUS neutron-radiation dose at pH 7.2. Error bars represent the standard errors of the means of three experiments. ■, without WR1065; ●, with WR1065.

protector on the elution kinetics of rejoining of DSB after exposure of the cells to a neutron dose of 100 Gy. In these experiments, V79 cells were irradiated without WR1065 and then allowed time to repair at 37°C, in either the presence or absence of the radioprotector. Rather than an apparent repair/rejoining process of damaged DNA (occurring as measured by neutral elution following high doses of JANUS neutrons), the DNA of irradiated cells appeared to degrade as a function of time over the first 180 min following irradiation. The presence of WR1065 during the post-irradiation incubation appears to retard this process, and a

**Figure 4** Double-strand scission factors (see text) describing the kinetics of rejoining of DNA breaks as a function of time following incubation at 37°C in the presence (■) or absence (●) of WR1065 after irradiation with JANUS neutrons (100 Gy). Error bars represent standard errors of the means of three experiments.

**Figure 5** Double-strand scission factors (see text) describing the kinetics of rejoining of DNA breaks as a function of time following incubation at 4°C in the presence (■) or absence (●) of WR1065 after irradiation with JANUS neutrons (100 Gy). Error bars represent standard errors of the means of three experiments.

rejoining of DSB as a function of time can be observed (see Figure 4). When this experiment was performed at 4°C, no evidence of DNA degradation was observed for cells incubated in either the absence or presence of WR1065 (see Figure 5). In addition, we observed no effect of holding cells at 4°C for up to 3 h on the formation of DSB in non-irradiated control cells.

### Discussion

Fission spectrum neutrons from the JANUS reactor are known to be significantly more clastogenic (Schwartz *et al.*, 1988), mutagenic (Grdina *et al.*, 1988), carcinogenic (Han & Elkind, 1979; Thomson *et al.*, 1982) and lethal (Ngo *et al.*, 1977) than $^{60}$Co $\gamma$-rays and/or X-rays. Consequently, it was of interest to assess whether similar differences could be detected at the level of DNA damage and/or repair as measured by the neutral filter elution technique at pH 7.2.

Clearly, the enhanced induction of these deleterious end-points by fission spectrum neutrons as compared to low-LET radiations cannot be accounted for by a concomitant enhanced frequency or number of neutron-induced DSB (e.g. we observed an RBE value of 1). Other investigators using either sucrose gradient sedimentation analysis (Furuno *et al.*, 1979) or a hydroxylapatite-DNA unwinding technique (Sakai *et al.*, 1987), or neutral elution (Prise *et al.*, 1987) have also reported RBE values of one for DSB induction by neutrons. Peak and his co-workers at the Argonne National Laboratory, using human P3 teratocarcinoma cells, have also observed an RBE of 1 with JANUS neutrons (personel communication, manuscript submitted). The observed differences in the relationship between low LET radiation-induced DSB and cell toxicity as compared to neutron-induced DSB and cell survival has led investigators to conclude that either there is no relationship between induced DSB and cell kill or there are qualitative differences in the DSB lesion produced by high as compared to low LET radiations (Prise *et al.*, 1987). These data, along with the results reported here, strongly suggest, therefore, that it is the quality or nature of the DSB lesion produced by JANUS neutrons that accounts for their enhanced deleterious effects.

This conclusion is further supported by the observation that WR1065, in contrast to its ability to protect against DSB formation by $^{60}$Co $\gamma$-rays by a factor of 1.7 (Sigdestad *et al.*, 1987), was unable to afford any protection against the induction of DSB by JANUS neutrons. The only effect of WR1065 observed on neutron-induced DSB lesions at very high radiation doses appeared to be related to post-irradiation DSB rejoining processes. The presence of WR1065 following irradiation appeared to inhibit the formation of additional DSB. These lesions were most probably enzymatically induced because their formation was affected not only by the presence of the radioprotector but also by temperature (i.e. 4°C). Aminothiols are known to affect enzymatic activity related to DNA synthesis and repair (LaSalle & Billen, 1964; Billen, 1983; Grdina & Nagy, 1986). Alternatively, the quality or nature of the DNA lesions formed by high doses of JANUS neutrons may have induced considerable nuclease activity as part of the 'repair' process. WR1065 can inhibit this activity. Under these circumstances, a small degree of DSB rejoining can be observed within the first 3 h following irradiation. Whether the inhibition by WR1065 of the post-irradiation formation of DSB is advantageous with respect to cellular repair at these high doses is unclear at present. However, reports that WR1065 can protect against JANUS-neutron-induced chromatid aberrations (Schwartz *et al.*, 1988) and mutagenesis (Grdina *et al.*, 1988) at much lower radiation doses suggest that it is capable of enhancing a post-irradiation repair process(es).

The mechanisms suggested to account for the radioprotective actions of aminothiols are numerous. They include the ability to scavenge free radicals (Hutchison, 1961), donate hydrogen atoms for chemical repair (Alexander & Charlesby, 1954), affect enzymatic systems involved in DNA synthesis and repair (LaSalle & Billen, 1964), bind to and stabilise chromatin material (Brown, 1967), and affect cell cycle progression (Grdina & Nagy, 1986). There is evidence to support each of these mechanisms, and the ultimate effect at the cellular level is probably the result of the integration of all or most of these mechanisms.

The authors wish to acknowledge G.L. Holmblad for dosimetry determinations and C. Fox for computer analysis of the data. The protector used throughout these experiments was kindly provided by Col. David E. Davidson, Jr., Director, Division of Experimental Therapeutics, Walter Reed Army Medical Center, Washington, DC, USA. The research was funded in part by the US Department of Energy under contract No. W-31-109-ENG-38 and in part by the Department of Health and Human Services, under NIH/NCI grant No. 5RO1 CA-37435.

### References

ALEXANDER, P. & CHARLESBY, A. (1954). Physico-chemical methods of protection against ionizing radiations. In *Radiobiology Symposium 1954*, Bacq, F. & Alexander, P. (eds) p. 49. Academic Press: New York.

APFFEL, C.A., WALKER, J.E. & ISSARESCU, S. (1975). Tumor rejection in experimental animals treated with radioprotective thiols. *Cancer Res.*, **35**, 429.

BILLEN, D. (1983). The effects of radioprotectors on DNA polymerase I-directed repair synthesis and DNA strand breaks in toluene-treated and x-irradiated *Escherichia coli*. *Radiat. Res.*, **95**, 158.

BRADLEY, M. O. & KOHN, K.W. (1979). X-ray induced DNA double strand break production and repair in mammalian cells as measured by neutral filter elution. *Nucleic Acid Res.*, **7**, 793.

BROWN, P.E. (1967). Mechanism of action of aminothiol radioprotectors. *Nature*, **213**, 363.

CORN, B.W., LIBER, H.L. & LITTLE, J.B. (1987). Differential effects of radical scavengers on x-ray-induced mutation and cytotoxicity in human cells. *Radiat. Res.*, **109**, 100.

FURUNO, I., YADA, T., MATSUDAIRA, H. & MAUYAMA, T. (1979). Induction and repair of DNA strand breaks in cultured mammalian cells following fast neutron irradiation. *Int. J. Radiat. Biol.*, **36**, 639.

GLOVER, D., GLICK, J.H., WEILER, C., YUHAS, J.M. & KLIGERMAN, M.M. (1984). Phase 1 trials of WR2721 and *cis*-platinum. *Int. J. Radiat. Oncol. Biol. Phys.*, **10**, 1781.

GRDINA, D.J. & NAGY, B. (1986). The effect of 2-[(aminopropyl)amino] ethanethiol (WR1065) on radiation-induced DNA damage and repair and cell progression in V79 cells. *Br. J. Cancer*, **54**, 933.

GRDINA, D.J., NAGY, B., HILL, C.K., WELLS, R.L. & PERAINO, C. (1985*a*). The radioprotector WR1065 reduces radiation-induced mutations at the hypoxanthine-guanine phosphoribosyl transferase locus in V79 cells. *Carcinogenesis*, **6**, 929.

GRDINA, D.J., NAGY, B. & SIGDESTAD, C.P. (1988). Radioprotectors in treatment therapy to reduce risk in secondary tumor induction. *Pharmacol. Ther.*, **39**, 21.

GRDINA, D.J., PERAINO, C., CARNES, B.A. & HILL, C.K. (1985*b*). Protective effect of S-2′-(3-aminopropylamino) ethylphosphorothioic acid against the induction of altered hepatocyte foci in rats treated once with gamma radiation within one day after birth. *Cancer Res.*, **45**, 5379.

HAN, A. & ELKIND, M.M. (1979). Transformation of mouse C3H/10T1/2 cells by single and fractionated doses of x-rays and fission-spectrum neutrons. *Cancer Res.*, **39**, 123.

HILL, C.K., NAGY, B., PERAINO, C. & GRDINA, D.J. (1986). 2-[(Aminopropyl)amino] ethanethiol (WR1065) is antineoplastic and antimutagenic when given during $^{60}$Co $\gamma$-ray irradiation. *Carcinogenesis*, **7**, 665.

HUTCHISON, F. (1961). Sulfhydryl groups and the oxygen effect on irradiated dilute solutions of enzymes and nucleic acids. *Radiat. Res.*, **14**, 721.

KLIGERMAN, M.M., GLOVER, D.J., TURRISI, A.T. and 6 others (1984). Toxicity of WR2721 administered in single and multiple doses. *Int. J. Radiat. Oncol. Biol. Phys.*, **10**, 1773.

LA SALLE, M. & BILLEN, D. (1964). Inhibition of DNA synthesis in murine bone-marrow cells by AET and cysteamine. *Ann. NY Acad. Sci.*, **114**, 622.

MARQUARDT, H., SAPOZINK, M.D. & ZEDECK, M.S. (1974). Inhibition by Cysteamine-HCl of oncogenesis induced by 7,12-dimethylbenz[a]anthracene without affecting toxicity. *Cancer Res.*, **34**, 3387.

MEYN, R.E. & JENKINS, W.T. (1983). Variation in normal and tumor tissue sensitivity of mice to ionizing radiation-induced DNA strand breaks *in vivo*. *Cancer Res.*, **43**, 5668.

MILAS, L., HUNTER, N., STEPHENS, C.L. & PETERS, L.J. (1984). Inhibition of radiation carcinogenesis by *S*-2-(3-aminopropylamino) ethylphosphorothioic acid. *Cancer Res.*, **44**, 5567.

MURRAY, D. VAN ANKEREN, S.C., MILAS, L. & MEYN, R.E. (1988). Radioprotective action of WR1065 on radiation-induced DNA strand breaks in cultured Chinese hamster ovary cells. *Radiat. Res.*, **113**. 155.

NAGY, B. DALE, P.J. & GRDINA, D.J. (1986). Protection against *cis*-diamminedichloroplatinum cytotoxicity and mutagenicity in V79 cells by 2-[(aminopropyl)amino]ethanethiol. *Cancer Res.*, **46**, 1132.

NAGY & GRDINA, D.J. (1986). Protective effects of 2-[(aminopropyl)amino] ethanethiol against bleomycin and nitrogen mustard-induced mutagenicity in V79 cells. *Int. J. Radiat. Onc. Biol. Phys.*, **12**, 1475.

NGO, F.Q.H., HAN, A., UTSUMI, H. & ELKIND, M.M. (1977). Comparative radiobiology of fast neutrons: Relevance to radiotherapy and basic studies. *Int. J. Radiat. Onc. Biol. Phys.*, **3**, 187.

PHILLIPS, T.L. (1980). Rationale for initial clinical trials and future development of radioprotectors. *Cancer Clin. Trials*, **3**, 165.

PRISE, K.M., DAVIES, S. & MICHAEL, B.D. (1987). The relationship between radiation-induced DNA double-strand breaks and cell kill in hamster V79 fibroblasts irradiated with 250 kVp x-rays, 2.3 MeV neutrons or $^{238}$Pu $\alpha$-particles. *Int. J. Radiat. Biol.*, **52**, 893.

PURDIE, J.W. (1979). A comparative study of the radioprotective effects of cysteamine, WR2721, and WR1065 in cultured human cells. *Radiat. Res.*, **77**, 303.

SAKAI, K., SUZUKI, S., NAKAMURA, N. & OKADA, S. (1987). Induction and subsequent repair of DNA damage by fast neutrons in cultured mammalian cells. *Radiat. Res.*, **110**, 311.

SAWADA, S. & OKADA, S. (1970). Cysteamine, cystamine and single-strand breaks of DNA in cultured mammalian cells. *Radiat Res.*, **44**, 116.

SCHWARTZ, J.L., GIOVANAZZI, S.M., KARRISON, T., JONES, C. & GRDINA, D.J. (1988). 2-[(aminopropyl)amino] ethanethiol-mediated reductions in $^{60}$Co $\gamma$-ray and fission-spectrum neutron-induced chromosome damage in V79 cells. *Radiat. Res.*, **113**, 145.

SIGDESTAD, C.P., TREACY, S.H., KNAPP, L.A. & GRDINA, D.J. (1987). The effect of 2-[(aminopropyl)amino] ethanethiol (WR1065) on radiation induced DNA double strand damage and repair in V79 cells. *Br. J. Cancer*, **55**, 477.

TAKEUCHI, I.K. & MURAKAMI, U. (1978). The preventive influence of cysteamine on the teratogenic action of 5-azacytidine. *Life Sci.*, **23**, 897.

THOMSON, J.F., LOMBARD, L.S., GRAHN, D., WILLIAMSON, F.S. & FRITZ, T.E. (1982). RBE of fission neutrons for life shortening and tumorigenesis. In *Radiation Protection: Neutron Carcinogenesis*, Broerse, J.J. & Gerber, G.B. (eds) p.75, EUR 8084 EN: Rijswijk, The Netherlands.

WILLIAMSON, F.S. & FRIGERIO, N. (1972). Field mapping and depth dosimetry in the Janus high flux irradiation room – a fast neutron facility for biological research. In *Proceedings of the First Symposium on Neutron Dosimetry in Biological and Medical Research*, Neuherberg, Munich, p. 743.

YUHAS, J.M. (1979). Differential protection of normal and malignant tissues against the cytotoxic effects of mechlorethamine. *Cancer Treat. Rep.*, **63**, 971.